# Pericapsular Nerve Group (PENG) Associated with Lateral Femoral Cutaneous Nerve (LFCN) Block Versus Fascia Iliaca Compartment Block (FICB) for Total Hip Replacement Surgery: Double-Blind Randomized Controlled Trial

**DOI:** 10.3390/jpm15060230

**Published:** 2025-06-03

**Authors:** Francesco Vetrone, Stefano Marelli, Andrea Galimberti, Michele Umbrello, Miriam Gotti, Angelo Pezzi, Alessandro Girombelli

**Affiliations:** 1 Department of Anesthesiology, Intensive Care and Pain Medicine, Policlinico di Monza, 20900 Monza, Italy; 2 Department of Anesthesiology and Critical Care Medicine, ASST Nord Milano, Bassini Hospital, 20092 Cinisello Balsamo, Italy; 3 Department of Medicine and Surgery, University of Milan-Bicocca, 20900 Monza, Italy; 4 Department of Anesthesiology, Intensive Care and Emergency Medicine, ASST Nord Milano, Sesto San Giovanni Hospital, 20099 Sesto San Giovanni, Italy; 5 Department of Intensive Care and Anesthesia, ASST Ovest Milanese, Ospedale Civile di Legnano, Via Giovanni Paolo II, 20025 Legnano, Italy; 6 Division of Anesthesiology, Department of Anesthesiology, Intensive Care and Emergency Medicine, “Ente Ospedaliero Cantonale” (EOC), Ospedale Regionale di Lugano, 69000 Lugano, Switzerland

**Keywords:** anesthesia, arthroplasty, hip replacement, nerve block, postoperative recovery, pain, opioids, ultrasound, personalized medicine

## Abstract

**Background:** Total hip arthroplasty (THA) improves the quality of life by alleviating pain and restoring function. The optimal pain control with minimal muscle weakness is paramount for early rehabilitation and for reducing complications. Although PROSPECT and ICAROS guidelines recommend the Fascia Iliaca Compartment Block (FICB), it is associated with insufficient pain relief and a prolonged quadriceps motor block. The association of the PENG (Pericapsular Nerve Group) with LFCN (lateral femoral cutaneous nerve) blocks may address these limitations, provide improved motor-sparing pain control, and offer a more tailored approach that enhances both an early postoperative recovery and patient satisfaction. **Methods:** A randomized controlled trial (November 2023–July 2024) compared the PENG + LFCN to the FICB in patients undergoing elective THAs under spinal anesthesia. The primary outcome was quadriceps weakness at 6 h post-block. Secondary outcomes included the degree of hip flexion and pain scores at 6, 24, and 48 h post-block, opioid consumption, and time to ambulation. **Results:** Fifty-eight patients were randomized (twenty-nine per group). The PENG + LFCN group achieved a significantly greater muscle strength (MRC: 4 [4; 4] vs. 3 [3; 4], *p* < 0.0001) and better hip flexion at all measured moments (6 h: 45° [37; 60] vs. 30° [25; 43], 24h: 59° [49; 66] vs. 47° [36; 50], 48 h: 62° [55; 70] vs. 50° [40; 55], all *p* < 0.0001). Resting pain was lower in the PENG + LFCN group (0 [0; 1], 0 [0; 2], and 0 [0; 1] vs. 2 [0; 3], 1 [0; 3], 1 [0; 3]), as was the dynamic pain during movement (1 [0; 2], 2 [2; 4], and 2 [1; 2] vs. 3 [2; 5], 3 [2; 4], and 3 [1; 3]; all *p* < 0.001), along with a lower total opioid consumption (0 [0; 0] vs. 7.5 [7.5; 22.5] MME, *p* < 0.001). **Conclusions:** The PENG + LFCN block outperformed the FICB in muscle strength, hip flexion, pain control, and opioid use, suggesting it may be a more effective option for THAs.

## 1. Introduction

The number of total hip arthroplasty (THA) procedures is ever growing since it offers a considerable improvement of the quality by promoting functional recovery and reducing chronic pain [1]. However, the complex innervation of the hip joint represents a significant challenge for the anesthesiologist managing perioperative pain [2]. Up to 23% of patients develop significant postoperative and chronic pain [3,4]. Additionally, a significant number of patients experience an intense postoperative pain that hinders the prompt initiation of physical rehabilitation [4]. Early postoperative rehabilitation and functional recovery are well known to be essential for reducing the risk of deep vein thrombosis and postoperative delirium, shortening the length of hospital stays, and decreasing perioperative mortality [5,6,7,8,9].

In THA, the surgical approach itself can significantly affect postoperative outcomes. Factors such as the operative time, soft tissue manipulation, and the extent of dissection influence pain levels and functional recovery. Common approaches include the direct anterior (DAA), lateral (LA), posterior (PA), and posterolateral (PLA), though no clear consensus exists on the most optimal technique. The DAA technique, due to its minimally invasive nature and intermuscular access, spares surrounding muscles and tissues, helping preserve the joint capsule integrity and hip stability. It facilitates early rehabilitation, reduces postoperative complications, and supports a faster functional recovery. A recent meta-analysis found that the DAA technique led to better early outcomes (within 1–3 months) and the shortest hospital stays, although it was associated with longer operative times compared to the PA approach [10]. While some studies have reported mixed results, evidence suggests that the DAA technique may also reduce blood loss and improve muscle perfusion, likely due to the reduced soft tissue trauma [11,12,13]. To minimize potential bias, this study analyzes only THA procedures performed using the same technique (DAA) and by the same surgical team. 

Various perioperative anesthetic and analgesic strategies have been described. The latest guidelines recommend the Fascia Iliaca Compartment Block (FICB) as the preferred peripheral nerve block to achieve effective analgesia with minimal side effects, thereby facilitating early postoperative mobility [14,15]. Despite this recommendation, several concerns have been raised regarding the FICB, such as its inadequate analgesia for the medial hip joint and prolonged quadriceps motor block [16,17]. This highlights the need for a different approach capable of achieving effective pain control without impairing functional recovery.

The Pericapsular Nerve Group (PENG) block is emerging as an innovative technique that may overcome the shortcomings of the FICB. The PENG block is widely recognized for its effectiveness in relieving hip fracture pain and ensuring adequate analgesia post-THA while preserving quadriceps strength, a critical factor for early postoperative mobilization [18,19,20,21,22]. However, this block does not adequately address the surgical incision site pain and thus requires an adjunctive technique, like local infiltration anesthesia (LIA), to improve pain management [23,24,25].

Several studies indicate that adding the LFCN block may enhance the effectiveness of the PENG block [26,27,28,29]. The LFCN runs along the sartorius and tensor fascia latae muscles, supplying sensory innervation to the skin over the anterior and lateral aspects of the thigh, from the hip to the knee, thus providing adequate coverage for surgical incision pain in THAs performed via the direct anterior approach (DAA) [30,31,32].

The combination of PENG and LFCN blocks represents a tailored approach that targets pain-generating nerves while minimizing the risk of side effects. Our hypothesis is that these blocks, when used together, can address both the hip joint pain (via the PENG block) and surgical site pain (via the LFCN block), while limiting the chance of unnecessary quadriceps weakness. This approach could be particularly beneficial for specific patient groups—such as those with chronic pain, obesity, or diabetes—where pain management can be especially challenging due to a higher likelihood of adverse events

Recently, we demonstrated that combining PENG and LFCN blocks improves the pain control and reduces opioid requirements [33]. In another study, our findings indicated benefits in terms of a reduced muscle weakness and lower opioid consumption [34].

The aim of this study was to further investigate the impact of these regional anesthesia techniques on postoperative muscle weakness.

## 2. Materials and Methods

We designed a randomized clinical trial to compare FICB with PENG + LFCN block for THA. After approval from the Local Ethics Committee (“Comitato Etico Territoriale Lombardia 3”) on 14 November 2023 (approval number 3703_PF_N), the trial was registered on ClinicalTrials.gov (Study ID: NCT06147401, principal investigator: Dr Francesco Vetrone) on 24 November 2023. The RCT was then conducted until July 2024. Written informed consent was obtained from the patients prior to enrolment. The relevant CONSORT guidelines were applied [35].

We tested the hypothesis that the PENG + LFCN block could provide better motor-sparing pain control than FICB. The primary outcome was the degree of residual motor weakness, assessed with the Medical Research Council Manual Muscle Testing scale (MRC—with scores ranging from 0 = no contraction to 5 = normal muscle strength) at 6 h postoperatively. The secondary outcomes were the degree of active hip flexion (a combined indicator of adequate muscle strength and pain control); effectiveness of perioperative analgesia, assessed with the Numerical Rating Scale (NRS); total opioid consumption; adverse events, such as nausea vomiting; and time to first ambulation.

All procedures were conducted in full compliance with the principles of the Declaration of Helsinki (revised October 2013) and adhered to Good Clinical Practice guidelines.

All patients considered for THA in the above-mentioned time frame were screened based on the following criteria.

This study included patients aged 18 years or older who were undergoing elective THA via DAA for non-traumatic hip disease. Participants were required to provide informed consent for spinal anesthesia and peripheral nerve block, either personally or through a legal guardian if appointed. Patients were excluded from the study if they were undergoing non-elective THA or if they lacked consent or refused to consent to the procedure. Other exclusion criteria included contraindications to neuraxial anesthesia, such as the following: signs suggestive of infection at the puncture site, an international normalized ratio (INR) greater than 1.5, activated partial thromboplastin time (aPTT) exceeding 1.5, or platelet counts below 40,000. Additionally, individuals with documented or suspected allergies to local anesthetics were also excluded from participation.

To ensure that the study population closely mirrors real-world clinical scenarios, patients with risk factors for developing chronic postoperative pain, such as those on chronic pain medication and individuals with diabetes mellitus, were not excluded from the study [36]. Patients with an American Society of Anesthesiologists (ASA) score of 3 or higher were also included in the study.

### 2.1. Randomization Process and Blinding Procedures

Eligible patients were randomly assigned to either the case group that received both PENG + LFCN blocks or the control group which received the FICB. Randomization was achieved using a computer-generated sequence in a 1:1 ratio based on patient admission order. To ensure the integrity of the study, multiple levels of masking were implemented, including assignment blinding and blinded outcome assessment.

A researcher not involved in postoperative evaluations conducted the group assignments, keeping both patients and the clinical team unaware of the treatment administered; only the anesthetist in the operating room knew the technique used. An independent investigator, unaware of the group assignment, collected all outcome data without interacting with the patient care team to prevent bias. Data analysis was also performed blindly, with the statistician receiving anonymized data, thus concealing the type of treatment administered.

### 2.2. Anesthetic Protocol

Each patient received subarachnoid neuraxial anesthesia with 0.5% hyperbaric bupivacaine (dosed at 0.06 mg per cm of height) and intrathecal sufentanil (5 mcg), administered in the lateral decubitus position, following the hospital’s protocol. All patients received either FICB or PENG + LFCN blocks, as determined by randomization.

Multimodal analgesia was implemented, consisting of 1 g acetaminophen and 400 mg ibuprofen administered every 8 h, they were started intravenously during surgery and continued orally postoperatively. Additionally, 8 mg of dexamethasone and 2 g of magnesium were administered intravenously as part of our hospital’s opioid sparing protocol. Oral opioids, specifically oxycodone, were reserved as rescue therapy for patients with an NRS score above 5, with dosing (starting at 5 mg) tailored to each patient’s needs and risk factors, in accordance with CDC guidelines [37]. No prophylactic antiemetics were administered.

The peripheral nerve blocks were performed immediately after neuraxial anesthesia to minimize patient discomfort and maintain blinding regarding the specific block given. These perineural blocks were conducted by experienced regional anesthesia operators under ultrasound guidance (Ecube i7, Alpinion, Biolive Group, Seoul, Republic of Korea). The spread of local anesthetic around the neural structures and into the surrounding fascia was evaluated to confirm the adequacy of the regional anesthesia technique. A 22 G, 50 mm or 80 mm echogenic needle (SonoPlex II PAJUNK^®^, 78187 Geisngen, Germany) was used, depending on the patient’s anthropometric characteristics, with an in-plane approach.

**Figure 1:**
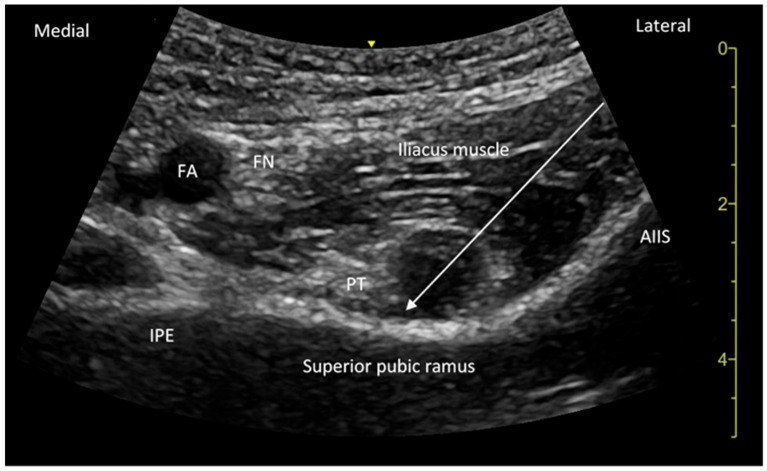
The sonoanatomy of the PENG Block. The needle is represented by the arrow. Once the needle is aligned with the plane of the iliopsoas tendon and the periosteum between the anterior inferior iliac spine (AIIS) and the iliopubic eminence (IPE), 20 mL of local anesthetic is injected, resulting in the elevation and displacement of the tendon. PT: psoas tendon, FA: femoral artery, IPE: iliopubic eminence, arrow: needle, and AIIS: anterior inferior iliac spine.

Regarding the PENG block, after disinfecting the skin, a 2–5 MHz curvilinear probe was positioned in the peri-inguinal region, aimed at the hip capsule and aligned with the pubic ramus. This setup allowed for clear visualization of key anatomical landmarks, including the anterior inferior iliac spine (AIIS), iliopubic eminence (IPE), psoas tendon, and femoral artery and vein. The needle was then advanced from lateral to medial until it made contact with the bone beneath the psoas tendon, ensuring that the tendon was not punctured. Subsequently, 20 mL of 0.5% ropivacaine was injected into the fascial plane between the psoas tendon and the superior pubic ramus (Figure 1).

**Figure 2:**
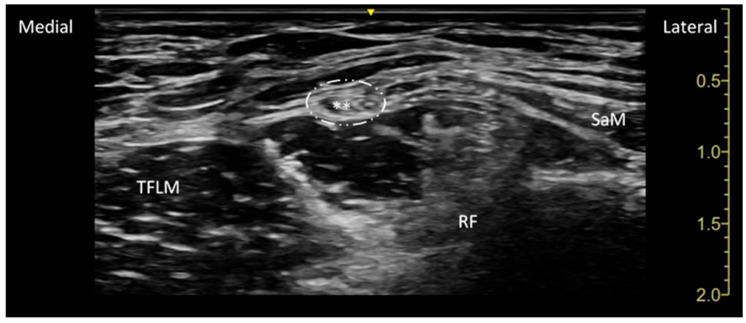
The sonoanatomy of the lateral femoral cutaneous nerve (LFCN) 10 cm distal to the Anterior Superior Iliac Spine (ASIS). The dashed lines indicate the so-called fat-filled flat tunnel (FFFT), the space that contains the lateral femoral cutaneous nerve (hyperechoic mass indicated by asterisks) between the sartorius muscle (SaM) and the tensor fascia latae muscle (TFLM). RF: rectus femoris muscle.

Following this, the LFCN block was performed. The LFCN is located in the subcutaneous plane between the sartorius muscle and the tensor fascia lata. A linear ultrasound probe (7.5–12 MHz) was used to guide the injection, with 10 mL of 0.5% ropivacaine injected into the fat-filled flat tunnel (FFFT) near the nerve, at the level of the superior anterior iliac spine (Figure 2).

**Figure 3:**
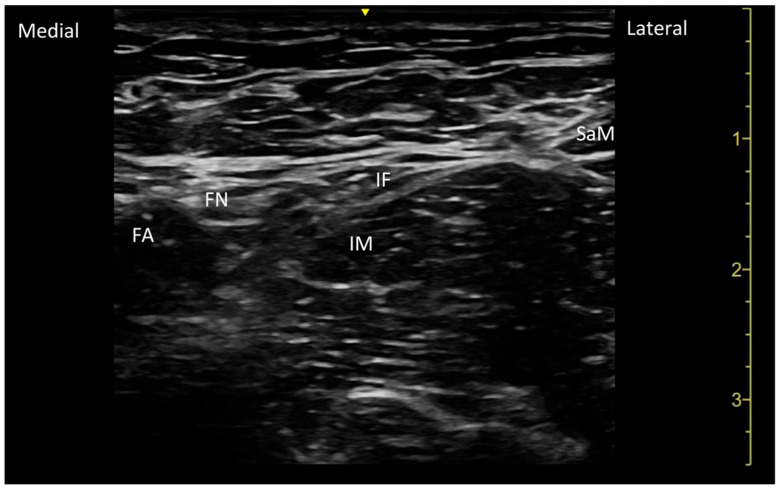
The sonoanatomy of the Fascia Iliaca Block (FICB). The block is performed approximately at the lateral third of the line connecting the Anterior Superior Iliac Spine (ASIS) and the pubic tubercle. FI: fascia iliaca; SaM: sartorius muscle, FN: Femoral Nerve, FA: femoral artery, and IM: iliopsoas muscle.

The patients in the control group received the FICB. After skin disinfection, the FICB was performed using a linear ultrasound probe (7.5–12 MHz) with a 22-gauge, 50 mm ultrasound-compatible needle. The needle was advanced above the fascia iliaca, and once the needle tip was positioned in the space between the iliopsoas muscle and the internal oblique muscle, 20 mL of 0.5% ropivacaine was injected (Figure 3).

To guarantee uniformity, all surgeries were conducted according to ERAS (Early Recovery After Surgery) guidelines by the same surgical and anesthesiology team.

### 2.3. Outcome Variables

The collected data included patient demographics such as age, sex, weight, height, BMI (Body Mass Index), ASA clinical status, and the presence of chronic pain medication usage or diabetes mellitus. Additional variables recorded included the degree of weakness or residual paresis of the quadriceps femoris muscle assessed using the MRC scale at 6, 24, and 48 h postoperatively. The degree of hip flexion on the operative side was measured using a Digital Angle Gauge in the supine position at 6, 24, and 48 h postoperatively, during active flexion.

Pain levels were evaluated using the NRS for both dynamics and static pain at the same time intervals. The time to first needed (PRN, Pro Re Nata) opioid request PRN, total opioid dose (expressed in Morphine Milligram Equivalent—MME), and any reported adverse events—such as nausea, vomiting, hypo- or paresthesia, hypotension, or falls—were also documented. All data were subsequently entered into an Excel spreadsheet (Microsoft Office, Redmond, DC, USA) for analysis.

### 2.4. Sample Size Estimation and Statistical Analysis

For the sample size calculation, due to the lack of similar studies at the time of design, we relied on results from our retrospective study comparing the FICB to the PENG block combined with the LFCN block [34]. The study indicated a significantly higher MRC score of the quadriceps muscle at 6 h post-surgery in the PENG + LFCN group compared to the FICB group (median MRC: 4 [4; 5] vs. 3 [2; 4], *p* < 0.0001)]. Based on these findings, we calculated a sample size of 24 patients per group to achieve 90% power, anticipating a 1-point difference on the MRC scale. To account for a projected dropout rate of 25%, a total sample size of 60 subjects was deemed appropriate (calculated using G*Power 3.1.9.7; Universität Kiel, DE).

Categorical variables were presented as absolute frequencies (n) and percentages (%). Continuous variables were expressed as mean and standard deviation if they followed a normal distribution (assessed by the Shapiro–Francia test) or as median and interquartile range (IQR, with 25th and 75th percentiles) if they did not.

Statistical analysis was performed using STATA 18.0 software (StataCorp LLC, College Station, TX, USA). Statistical significance was set at *p* < 0.05 for all comparisons. Fisher’s exact test was employed for categorical variables, while Student’s *t*-test was used for normally distributed continuous variables. For non-normally distributed continuous variables, the Wilcoxon–Mann–Whitney rank-sum test was applied.

Considering the nature of the study design and after verifying the absence of outliers in the repeated measures and the balance in the number of measures for each experimental unit, the analysis of repeated measures over time was conducted using two-way ANOVA. This method was employed to compare variables between the two study groups over time, with the type of block (inter-subject variable) and time (intra-subject variable) treated as independent factors. The interaction between block type and time was also analyzed, with significant corrections made using the Greenhouse–Geisser method. In cases of significant interaction between variables, the Siegel–Tukey Test was employed for multiple comparisons. Three *p*-values were reported: P (block) for group effects, P (time) for time effects, and P (block*time) for the interaction between group and time effects.

## 3. Results

**Figure 4:**
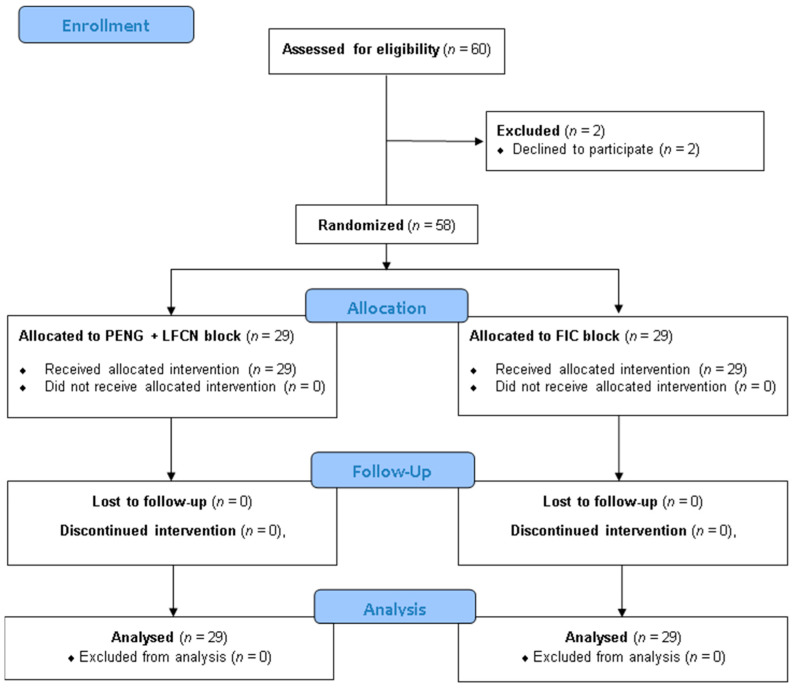
CONSORT flow diagram. CONSORT indicates Consolidated Standards of Reporting Trials [35].

Sixty adults scheduled for THAs were recruited for this study from November 2023 to July 2024. Two eligible patients declined to participate, resulting in a final sample of 58 patients, as illustrated in the CONSORT diagram (Figure 4).

No statistically significant differences were observed between the FICB and PENG + LFCN groups regarding demographic or clinical variables (Table 1).

### 3.1. Motor Function

The combination of PENG + LFCN blocks resulted in significantly less residual motor weakness, as indicated by a higher MRC score compared to the FICB group. Specifically, at 6 h postoperatively, the PENG + LFCN group had a significantly higher median MRC score (4 [4; 4] vs. 3 [3; 4], *p* < 0.0001).

This difference persisted at subsequent time points (24 and 48 h). Overall, MRC scores increased over time in both groups (p for time = 0.0304). However, the interaction between the block type and time on MRC scores was not statistically significant (p for interaction = 0.0777), suggesting that the impact of the block type on the MRC scores remained consistent over time (Table 2).

### 3.2. Hip Flexion

Similarly, the PENG + LFCN group demonstrated a significantly greater maximum degree of hip flexion at all measured time points. At 6 h, the PENG + LFCN group showed a median hip flexion of 45° [37; 60], compared to 30° [25; 43] in the FICB group (*p* < 0.0001). This trend was consistent at 24 and 48 h, with median flexion values of 59° [49; 66] vs. 47° [36; 50] and 62° [55; 70] vs. 50° [40; 55], respectively (*p* < 0.0001 at all time points). The effect of the block type on hip flexion was statistically significant (*p* < 0.0001), as was the effect of time (*p* < 0.0001). However, the interaction between the block type and time was not significant (*p* = 0.7405), indicating that the relative difference in hip flexion between the two groups remained consistent throughout the study period (Table 2).

### 3.3. Time to Ambulation

Regarding the time to ambulation, while the PENG + LFCN group showed a tendency for earlier mobilization (22 h [20; 24] vs. 24 h [22; 24]), no statistically significant differences were observed between the two groups (*p* = 0.0564).

### 3.4. Pain Outcomes

The PENG + LFCN group experienced significantly less static pain compared to the FICB group (*p* < 0.0001). At 6 h, the median static NRS score for the PENG + LFCN group was 0 [0; 1], while the FICB group reported a median score of 2 [0; 3]. No significant changes in static NRS scores were observed over time (p for time = 0.4825), indicating that static pain levels remained stable throughout the assessed time points. Additionally, the interaction between the block type and time was not significant (*p* = 0.3585), suggesting that the effect of the block type on static pain did not change. The dynamic NRS scores also favored the PENG + LFCN group, with significantly lower pain levels compared to the FICB group (*p* = 0.0001). At 6 h, the median dynamic NRS score for the PENG + LFCN group was one [0; 2], compared to three [2; 5] for the FICB group. Unlike static the NRS, dynamic pain levels varied significantly over time, peaking at 24 h (p for time < 0.0001). However, the interaction between the block type and time for the dynamic NRS was not significant (*p* = 0.1029), indicating that the relative difference in the dynamic pain between the two groups remained consistent over time (Table 2).

### 3.5. Opioid Consumption and PONV

The total opioid consumption was higher in the FICB group compared to the PENG + LFCN group, with a median MME of 7.5 at a median time of 12 [10; 16] hours post-block (7.5 [7.5; 22.5] vs. 0 [0; 0], *p* < 0.001). As a result, no comparisons could be made regarding the time to the first rescue opioid. Additionally, the incidence of postoperative nausea and vomiting (PONV) was notably lower in the PENG + LFCN group, where no cases were observed, compared to a 13.8% incidence in the FICB group (*p* = 0.0383).

## 4. Discussion

The results of this single-center RCT show the following: (i) the combination of PENG and LFCN blocks leads to greater muscle strength compared to the FICB at all three time points; (ii) the PENG + LFCN group demonstrates improved hip movement relative to the FICB group throughout the study period; (iii) enhanced pain control is observed in the PENG + LFCN group across all measured time points; (iv) there is no time-dependent interaction, indicating that the advantages of the dual PENG and LFCN block are stable over time; (v) the intervention group exhibits a reduced need for rescue opioids; and (vi) a potentially lower incidence of PONV is noted in the intervention group, although the study was underpowered for this secondary endpoint. Effective analgesia and prompt postoperative rehabilitation are crucial for patients undergoing THAs. Early mobilization enhances joint movement, minimizes soft tissue swelling, reduces the need for further analgesia, and it could shorten the length of hospital stays [6,7,8,38].

The sample size calculation was initially performed assuming a conservative 25% dropout rate, with 24 patients per group considered sufficient to achieve a statistical power of 90%. However, the study was concluded after enrolling 58 patients, as some patients withdrew. Although this number is lower than originally calculated, it does not result in significant changes to the statistical power, as the dropout rate was lower than expected and the final sample size still exceeds the minimum required (i.e., 24). In fact, the a posteriori calculation of the study’s statistical power, with 58 patients, is 95.4%.

While the PENG block is recognized for sparing quadriceps strength, a recent RCT comparing the PENG + LFCN with the S-FICB (suprainguinal iliac fascia compartment block) suggests this benefit is primarily evident only within 6 h following a THA performed using a posterolateral approach [39]. In contrast, our data show that this motor-sparing effect persists over time, aligning with findings by Liu [40]. Although comparisons are limited due to several key differences, such as the surgical approach, the use of general anesthesia instead of neuraxial, different anesthetic concentrations (Liang used 30 mL of 0.33% ropivacaine, Liu 40 mL of 0.375%), and the use of the S-FICB instead of the FICB, these findings imply that the PENG + LFCN may improve patient satisfaction, enable more intensive hip rehabilitation, and enhance the range of motion, and therefore could contribute to improved long-term functional outcomes [39,40].

While some authors have utilized lower concentrations of local anesthetic [29,41], we opted for 0.5% ropivacaine to extend the duration of the nerve block. The total dosage of the local anesthetic along with its absorption rate plays a crucial role in determining the effectiveness and longevity of the block [42,43,44].

A notable secondary outcome suggested that the PENG + LFCN group achieved better pain control, both at rest and during dynamic activities. Although the difference in pain scores was modest, it retained clinical significance considering that the minimal clinically important difference (MCID) for pain reduction is a reduction of 17 mm (IQR: 14–23) on a 0–100 mm visual analog scale (VAS) [45].

It is noteworthy that the pain reduction remains consistent over time, extending beyond the initial hours post-surgery. This result is in contrast with the findings of a recent systematic review by Ke et al., which found no evidence of the PENG block’s effectiveness at 48 h [18].

Furthermore, the reduction in pain experienced by the PENG + LFCN group correlated with a decrease in opioid consumption, both in terms of the cumulative dosage and the timing of requests. Although the reduction in the opioid requirement did not meet the clinician-perceived MCID of 10 Morphine Milligram Equivalents (MMEs) reported by Laigaard [45], we believe that every milligram of conserved morphine can decrease the risk of complications and improve discharge eligibility [46].

Postoperative opioid use can be considered as an indirect measure of the overall pain burden, therefore a lower opioid requirement during the postoperative phase may correlate with an improved postoperative experience.

These findings suggest that the addition of the LFCN block may provide effective analgesic coverage for the surgical incision of THA via the DAA. However, this contrasts with Pascarella et al., who found no pain improvement with the LFCN block compared to the wound infiltration in posterior THAs [47]. In their approach, the skin incision is innervated by the iliohypogastric nerve and the subcostal nerve, neither of which is adequately covered by the combination block.

Nonetheless, it remains unclear whether the differences observed in our study can be solely attributed to the effects of the LFCN, as the influence of the PENG block may also play a significant role.

The pain reduction observed in the PENG + LFCN group was associated with a significantly greater hip flexion at all time points. These data suggest a dual benefit in terms of reduced quadriceps weakness and improved pain control, both of these would be crucial for facilitating proper hip movement.

Despite the significant difference in the dynamic NRS between the two groups and although the PENG + LFCN group started walking slightly earlier, this difference did not reach statistical significance, showing comparable recovery times.

### Limitations

Our study has several limitations: First, the follow-up period was restricted to the duration of the hospital stay, which limited our ability to assess long-term outcomes after discharge. Additionally, the multimodal perioperative analgesia protocols implemented at our center may differ from those used at other institutions, potentially limiting the generalizability of our findings. The relatively small sample size was another constraint, as it limited our capacity to detect differences in secondary outcomes.

Another limitation is the imbalance in the dosage of the local anesthesia across the different peripheral nerve block groups. This choice is supported by the considerable heterogeneity of published trials and takes into consideration the originally described local anesthetic volume for these blocks. The dosages were chosen to achieve the optimal local anesthetic spread and block duration for each group, with the goal of optimizing the analgesic efficacy while minimizing the risk of muscle weakness.

Moreover, we did not employ quantitative sensory testing, such as a dynamometer, to quantify the quadriceps muscle strength and to objectively evaluate the effectiveness of the nerve block in assessing sensory loss. Lastly, the first postoperative ambulation had to be conducted under the supervision of the rehabilitation physicians, which may cause delays between their arrival and the patient’s ability to walk, potentially affecting the timing of mobility exercises.

Overall, while our findings provide valuable insights, these limitations highlight the need for further research to confirm our results and explore the long-term effects of the interventions.

## 5. Conclusions

In this study, the LFCN+PENG provided effective motor-sparing analgesia following the anterior approach THA. Patients in the PENG + LFCN group showed significantly higher MRC scores, indicating better preserved muscle strength, and achieved a greater hip range of motion compared to the FICB group. Additionally, this group reported lower NRS scores for pain at rest and during activities, highlighting the analgesic efficacy of the dual blocks.

Moreover, the PENG + LFCN group had a reduced opioid consumption, with no patients requiring rescue opioids. This reduction not only reflects the effectiveness of the nerve blocks in managing pain but also suggests a lower risk of opioid-related complications, thereby enhancing patient safety and satisfaction. Overall, the findings indicate that combining LFCN and PENG blocks may improve pain control and functional recovery following an anterior approach THA, contributing to a more effective and personalized patient-centered recovery process. While the results are promising, limitations, such as a short follow-up period, warrant further research to confirm these benefits across different settings and to assess long-term outcomes.

## Figures and Tables

**Table 1:** Case mix.

Variable	FICB (*n* = 29)	PENG + LFCN (*n* = 29)	Standardized Difference	*p*
Age (years)	71 ± 9	71 ± 10	0.0319	0.9053
Body weight (kg)	78.4 ± 16.6	79.8 ± 14.3	−0.0868	0.7423
Height (cm)	164.9 ± 8.9	169.1 ± 7.3	−0.5028	0.0606
BMI (kg/m^2^)	28.8 ± 5.7	27.8 ± 3.9	0.1196	0.4065
Male sex	14 (48.2%)	19 (65.5%)	−0.1474	0.1850
ASA status	2 [2; 2]	2 [2; 2]	0.0001	0.8283
Chronic pain medication	2 (6.9%)	3 (10.3%)	−0.1209	0.6402
Diabetes mellitus	5 (17.2%)	6 (20.7%)	−0.0865	0.7381
Italian nationality	29 (100%)	29 (100%)	0.0001	>0.9999

Case mix with standardized difference. PENG: Pericapsular Nerve Group, LFCN: lateral femoral cutaneous nerve, FICB: Fascia Iliaca Compartment Block, BMI: Body Mass Index, and ASA status: American Society of Anesthesiologists Physical Status Classification System.

**Table 2:** Outcomes.

Variable	FICB (*n* = 29)	PENG + LFCN (*n* = 29)	P (Group)	P (Time)	P (Interaction)
**MRC scale**			<0.0001	0.0304	0.0777
**6 h**	3 [3; 4]	4 [4; 4]			
**24 h**	4 [3; 4]	4 [4; 4]			
**48 h**	4 [3; 4]	4 [4; 5]			
**Degree of hip flexion**			<0.0001	<0.0001	0.7405
**6 h**	30 [25; 43]	45 [37; 60]			
**24 h**	47 [36; 50]	59 [49; 66]			
**48 h**	50 [40; 55]	62 [55; 70]			
**Static NRS**			<0.0001	0.4825	0.3585
**6 h**	2 [0; 3]	0 [0; 1]			
**24 h**	1 [0; 3]	0 [0; 2]			
**48 h**	1 [0; 3]	0 [0; 1]			
**Dynamic NRS**			0.0001	0.0381	0.1029
**6 h**	3 [2; 5]	1 [0; 2]			
**24 h**	3 [2; 4]	2 [2; 4]			
**48 h**	3 [1; 3]	2 [1; 2]			

Outcomes: the evaluation of the quadriceps femoris muscle strength recovery, hip flexion, and pain control. A two-way ANOVA was used to compare variables between study groups over time, with the block type (inter-subject) and time (intra-subject) as factors. Interactions were assessed with Greenhouse–Geisser corrections and analyzed using the Siegel-Tukey test. *p*-values for group effects (P (block)), time effects (P (time)), and their interaction (P (block*time)) are reported. PENG: Pericapsular Nerve Group, LFCN: lateral femoral cutaneous nerve, FICB: Fascia Iliaca Compartment Block, MRC: Medical Research Council Scale for Muscle Strength, and NRS: Numerical Rating Scale.

## Data Availability

Data will be available upon reasonable request to the corresponding author.

## Abbreviations

The following abbreviations are used in this manuscript:

THA	Total hip arthroplasty
PENG	Pericapsular Nerve Group
LFCN	Lateral femoral cutaneous nerve
FICB	Fascia Iliaca Compartment Block
RCT	Randomized clinical trial
PROSPECT	Procedure-Specific Postoperative Pain Management
ICAROS	International Consensus on Anaesthesia-Related Outcomes after Surgery group
INR	International normalized ratio
aPTT	Activated partial thromboplastin time
ASA	American Society of Anesthesiologists
AIIS	Anterior inferior iliac spine
IPE	Iliopubic eminence
FFFT	Fat-filled flat tunnel
SaM	Sartorius muscle
TFLM	Tensor fascia latae muscle
RF	Rectus Femoris Muscle.
MRC	Medical Research Council Scale for Muscle Strength
NRS	Numerical Rating Scale
ERAS	Early Recovery After Surgery
BMI	Body Mass Index
PRN	Pro Re Nata
MME	Morphine Milligram Equivalent
PONV	Postoperative nausea and vomiting
MCID	Minimal clinically important difference
VAS	Visual analog scale
DAA	Direct anterior approach

## References

[1] Maradit Kremers H., Larson D.R., Crowson C.S., Kremers W.K., Washington R.E., Steiner C.A., Jiranek W.A., Berry D.J. (2015). Prevalence of Total Hip and Knee Replacement in the United States. J. Bone Jt. Surg.-Am. Vol..

[2] Short A.J., Barnett J.J.G., Gofeld M., Baig E., Lam K., Agur A.M., Peng P.W. (2017). Anatomic Study of Innervation of the Anterior Hip Capsule. Reg. Anesth. Pain Med..

[3] Judge A., Arden N.K., Batra R.N., Thomas G., Beard D., Javaid M.K., Cooper C., Murray D., Exeter Primary Outcomes Study (EPOS) group (2013). The association of patient characteristics and surgical variables on symptoms of pain and function over 5 years following primary hip-replacement surgery: A prospective cohort study. BMJ Open.

[4] Beswick A.D., Wylde V., Gooberman-Hill R., Blom A., Dieppe P. (2012). What proportion of patients report long-term pain after total hip or knee replacement for osteoarthritis? A systematic review of prospective studies in unselected patients. BMJ Open.

[5] Richebé P., Capdevila X., Rivat C. (2018). Persistent Postsurgical Pain: Pathophysiology and Preventative Pharmacologic Considerations. Anesthesiology.

[6] Aprisunadi, Nursalam N., Mustikasari M., Ifadah E., Hapsari E.D. (2023). Effect of Early Mobilization on Hip and Lower Extremity Postoperative: A Literature Review. SAGE Open Nurs..

[7] Zhu S., Qian W., Jiang C., Ye C., Chen X. (2017). Enhanced recovery after surgery for hip and knee arthroplasty: A systematic review and meta-analysis. Postgrad. Med. J..

[8] Okamoto T., Ridley R.J., Edmondston S.J., Visser M., Headford J., Yates P.J. (2016). Day-of-Surgery Mobilization Reduces the Length of Stay After Elective Hip Arthroplasty. J. Arthroplast..

[9] Gan T.J. (2017). Poorly controlled postoperative pain: Prevalence, consequences, and prevention. J. Pain Res..

[10] Ang J.J.M., Onggo J.R., Stokes C.M., Ambikaipalan A. (2023). Comparing direct anterior approach versus posterior approach or lateral approach in total hip arthroplasty: A systematic review and meta-analysis. Eur. J. Orthop. Surg. Traumatol..

[11] Barrett W.P., Turner S.E., Murphy J.A., Flener J.L., Alton T.B. (2019). Prospective, Randomized Study of Direct Anterior Approach vs Posterolateral Approach Total Hip Arthroplasty: A Concise 5-Year Follow-Up Evaluation. J. Arthroplast..

[12] Jin M.W., Zhang L., Chu X.B., Lv S.J., Zhang J.J., Tong P.J., Pan Y. (2023). Comparison of clinical efficacy between direct anterior approach and posterolateral approach in primary total hip arthroplasty. Eur. Rev. Med. Pharmacol. Sci..

[13] Fransen B., Hoozemans M., Vos S. (2016). Direct anterior approach versus posterolateral approach in total hip arthroplasty: One surgeon, two approaches. Acta Orthop. Belg..

[14] Memtsoudis S.G., Cozowicz C., Bekeris J., Bekere D., Liu J., Soffin E.M., Mariano E.R., Johnson R.L., Hargett M.J., Lee B.H. (2019). Anaesthetic care of patients undergoing primary hip and knee arthroplasty: Consensus recommendations from the International Consensus on Anaesthesia-Related Outcomes after Surgery group (ICAROS) based on a systematic review and meta-analysis. Br. J. Anaesth..

[15] Anger M., Valovska T., Beloeil H., Lirk P., Joshi G.P., Van de Velde M., Raeder J., PROSPECT Working Group* and the European Society of Regional Anaesthesia and Pain Therapy (2021). PROSPECT guideline for total hip arthroplasty: A systematic review and procedure-specific postoperative pain management recommendations. Anaesthesia.

[16] Gasanova I., Alexander J.C., Estrera K., Wells J., Sunna M., Minhajuddin A., Joshi G.P. (2019). Ultrasound-guided suprainguinal fascia iliaca compartment block versus periarticular infltration for pain management after total hip arthroplasty: A randomized controlled trial. Reg. Anesth. Pain Med..

[17] Eshag M.M.E., Hasan L.O.M., Elshenawy S., Ahmed M.S., Mostafa A.E.-M.E., Abdelghafar Y.A., Althawadi Y.J., Ibraheem N.M., Badr H., AbdelQadir Y.H. (2024). Fascia iliaca compartment block for postoperative pain after total hip arthroplasty: A systematic review and meta-analysis of randomized controlled trials. BMC Anesthesiol..

[18] Ke J., Yang Y., Cao Y., Wang Y., Lin C. (2024). Efficacy and safety of pericapsular nerve group block in total hip arthroplasty: A meta-analysis and systematic review. Minerva Anestesiol..

[19] She C., Liu H. (2024). The efficacy of pericapsular nerve group block for reducing pain and opioid consumption after total hip arthroplasty: A systematic review and meta-analysis. J. Orthop. Surg. Res..

[20] Zheng J., Du L., Chen G., Zhang L., Deng X., Zhang W. (2023). Efficacy of pericapsular nerve group (PENG) block on perioperative pain management in elderly patients undergoing hip surgical procedures: A protocol for a systematic review with meta-analysis and trial sequential analysis. BMJ Open.

[21] Farag A., Hendi N.I., Diab R.A. (2023). Does pericapsular nerve group block have limited analgesia at the initial post-operative period? Systematic review and meta-analysis. J. Anesth..

[22] Morrison C., Brown B., Lin D.Y., Jaarsma R., Kroon H. (2021). Analgesia and anesthesia using the pericapsular nerve group block in hip surgery and hip fracture: A scoping review. Reg. Anesth. Pain Med..

[23] Lin D.Y., Brown B., Morrison C., Fraser N.S., Chooi C.S.L., Cehic M.G., McLeod D.H., Henningsen M.D., Sladojevic N., Kroon H.M. (2022). The Pericapsular nerve group (PENG) block combined with local infltration analgesia (LIA) compared to placebo and LIA in hip arthroplasty surgery: A multi-center double-blinded randomized-controlled trial. BMC Anesthesiol..

[24] Hu J., Wang Q., Hu J., Kang P., Yang J. (2023). Efficacy of Ultrasound-Guided Pericapsular Nerve Group (PENG) Block Combined with Local Infiltration Analgesia on Postoperative Pain after Total Hip Arthroplasty: A Prospective, Double-Blind, Randomized Controlled Trial. J. Arthroplast..

[25] Bravo D., Aliste J., Layera S., Fernández D., Erpel H., Aguilera G., Arancibia H., Barrientos C., Wulf R., León S. (2023). Randomized clinical trial comparing pericapsular nerve group (PENG) block and periarticular local anesthetic infiltration for total hip arthroplasty. Reg. Anesth. Pain Med..

[26] Roy R., Agarwal G., Pradhan C., Kuanar D. (2019). Total postoperative analgesia for hip surgeries, PENG block with LFCN block. Reg. Anesth. Pain Med..

[27] Kim D.H., Hong G., Lin E., Kim S.J., Beathe J., Wetmore D., Liu J. (2024). Combined Pericapsular Nerve Group Block and Intrapelvic Lateral Femoral Cutaneous Nerve Block Is Associated with Decreased Opioid Consumption After Hip Arthroscopy: A Retrospective Cohort Study. HSS J..

[28] Gurbuz H., Okmen K., Gultekin A. (2021). Postoperative pain management in patients with coxarthrosis undergoing total hip arthroplasty: PENG block combined with LFCN block or wound infiltration?. Minerva Anestesiol..

[29] Yoo S.H., Lee M.J., Beak M.H., Kim W.J. (2024). Efficacy of Supplemental Ultrasound-Guided Pericapsular Nerve Group (PENG) Block Combined with Lateral Femoral Cutaneous Nerve Block in Patients Receiving Local Infiltration Analgesia after Hip Fracture Surgery: A Prospective Randomized Controlled Trial. Medicina.

[30] Hopkins P.M., Ellis F.R., Halsall P.J. (1991). Evaluation of local anesthetic blockade of the lateral femoral cutaneous nerve. Anaesthesia.

[31] Corujo A., Franco C.D., Williams J.M. (2012). The sensory territory of the lateral cutaneous nerve of the thigh as determined by anatomic dissections and ultrasound-guided blocks. Reg. Anesth. Pain Med..

[32] Bergin P.F., Unger A.S. (2011). Direct Anterior Total Hip Arthroplasty. JBJS Essent. Surg. Tech..

[33] Girombelli A., Vetrone F., Saglietti F., Galimberti A., Fusaro A., Umbrello M., Pezzi A. (2024). Pericapsular nerve group block and lateral femoral cutaneous nerve block versus fascia iliaca block for multimodal analgesia after total hip replacement surgery: A retrospective analysis. Saudi J. Anaesth..

[34] Vetrone F., Saglietti F., Galimberti A., Pezzi A., Umbrello M., Cuttone G., La Via L., Vetrugno L., Deana C., Girombelli A. (2025). Pericapsular Nerve Group Block Plus Lateral Femoral Cutaneous Nerve Block vs. Fascia Iliaca Compartment Block in Hip Replacement Surgery. J. Clin. Med..

[35] Hopewell S., Chan A.W., Collins G.S., Hróbjartsson A., Moher D., Schulz K.F., Tunn R., Aggarwal R., Berkwits M., Berlin J.A. (2025). CONSORT 2025 statement: Updated guideline for reporting randomised trials. BMJ.

[36] Zhang B., Rao S., Mekkawy K.L., Rahman R., Sarfraz A., Hollifield L., Runge N., Oni J.K. (2023). Risk factors for pain after total hip arthroplasty: A systematic review. Arthroplasty.

[37] Dowell D., Ragan K.R., Jones C.M., Baldwin G.T., Chou R. (2022). CDC Clinical Practice Guideline for Prescribing Opioids for Pain—United States, 2022. MMWR Recomm. Rep..

[38] Gulotta L.V., Padgett D.E., Sculco T.P., Urban M., Lyman S., Nestor B.J. (2011). Fast track THR: One hospital’s experience with a 2-day length of stay protocol for total hip replacement. HSS J..

[39] Liang L., Zhang C., Dai W., He K. (2023). Comparison between pericapsular nerve group (PENG) block with lateral femoral cutaneous nerve block and supra-inguinal fascia iliaca compartment block (S-FICB) for total hip arthroplasty: A randomized controlled trial. J. Anesth..

[40] Liu M., Gao M., Hu Y., Ren X., Li Y., Gao F., Dong J., Dong J., Wang Q. (2024). Comparison of the Effect of Pericapsular Nerve Group Block Combined with Lateral Femoral Cutaneous Nerve Block and Fascia Iliaca Compartment Block in Patients Undergoing Hip Arthroscopy Under General Anesthesia: A Randomized, Double-Blind Trial. J. Pain Res..

[41] Pascarella G., Costa F., Del Buono R., Pulitanò R., Strumia A., Piliego C., De Quattro E., Cataldo R., Agrò F.E., Carassiti M. (2021). Impact of the pericapsular nerve group (PENG) block on postoperative analgesia and functional recovery following total hip arthroplasty: A randomised, observer-masked, controlled trial. Anaesthesia.

[42] Taylor A., McLeod G. (2020). Basic pharmacology of local anaesthetics. BJA Educ..

[43] Shigeta H., Yasumura R., Kotake Y. (2022). Comparison of plasma levobupivacaine concentrations with and without epinephrine following erector spinae plane block for breast cancer surgery: A randomized controlled trial. BMC Anesthesiol..

[44] De Cassai A., Bonanno C., Padrini R., Geraldini F., Boscolo A., Navalesi P., Munari M. (2021). Pharmacokinetics of lidocaine after bilateral ESP block. Reg. Anesth. Pain Med..

[45] Laigaard J., Pedersen C., Rønsbo T.N., Mathiesen O., Karlsen A.P.H. (2021). Minimal clinically important differences in randomised clinical trials on pain management after total hip and knee arthroplasty: A systematic review. Br. J. Anaesth..

[46] Dizdarevic A., Farah F., Ding J., Shah S., Bryan A., Kahn M., Kaye A.D., Gritsenko K. (2019). A Comprehensive Review of Analgesia and Pain Modalities in Hip Fracture Pathogenesis. Curr. Pain Headache Rep..

[47] Pascarella G., Costa F., Strumia A., Ruggiero A., Remore L.M., Lanteri T., Hazboun A., Longo F., Gargano F., Schiavoni L. (2024). Lateral Femoral Cutaneous Nerve Block or Wound Infiltration Combined with Pericapsular Nerve Group (PENG) Block for Postoperative Analgesia following Total Hip Arthroplasty through Posterior Approach: A Randomized Controlled Trial. J. Clin. Med..

